# Quality of life, disease activity and preferences for administration routes in rheumatoid arthritis: a multicentre, prospective, observational study

**DOI:** 10.1093/rap/rkac071

**Published:** 2022-09-02

**Authors:** Haner Direskeneli, Omer Karadag, Askin Ates, Abdurrahman Tufan, Nevsun Inanc, Serdar S Koca, Gozde Y Cetin, Servet Akar, Muhammet Cinar, Sedat Yilmaz, Neslihan Yilmaz, Ediz Dalkilic, Cemal Bes, Baris Yilmazer, Ali Sahin, Duygu Ersözlü, Mehmet E Tezcan, Nesrin Sen, Gokhan Keser, Umut Kalyoncu, Berkan Armagan, Basak Hacibedel, Kerem Helvacioglu, Teoman Y Cesur, Canberk S Basibuyuk, Serdar Alkan, Levent Mert Gunay

**Affiliations:** Division of Rheumatology, Department of Internal Medicine, Faculty of Medicine, Marmara University, Istanbul, Turkey; Division of Rheumatology, Department of Internal Medicine, Faculty of Medicine, Hacettepe University, Ankara, Turkey; Division of Rheumatology, Department of Internal Medicine, Faculty of Medicine, Ankara University, Ankara, Turkey; Division of Rheumatology, Department of Internal Medicine, Faculty of Medicine, Gazi University, Ankara, Turkey; Division of Rheumatology, Department of Internal Medicine, Faculty of Medicine, Marmara University, Istanbul, Turkey; Division of Rheumatology, Department of Internal Medicine, Faculty of Medicine, Fırat University, Elazıg, Turkey; Division of Rheumatology, Department of Internal Medicine, Faculty of Medicine, Kahramanmaras Sutcu Imam University, Kahramanmaras, Turkey; Division of Rheumatology, Department of Internal Medicine, Faculty of Medicine, Izmir Katip Celebi University, Izmir, Turkey; Clinic of Rheumatology, Gulhane Faculty of Medicine, Gulhane Training and Research Hospital, Health Science University, Ankara, Turkey; Clinic of Rheumatology, Gulhane Faculty of Medicine, Gulhane Training and Research Hospital, Health Science University, Ankara, Turkey; Division of Rheumatology, Department of Internal Medicine, Faculty of Medicine, TC Demiroglu Bilim University, Istanbul, Turkey; Division of Rheumatology, Department of Internal Medicine, Faculty of Medicine, Uludag University, Bursa, Turkey; Clinic of Rheumatology, Department of Internal Medicine, Istanbul Provincial Health Directorate, Basaksehir Cam and Sakura City Hospital, Istanbul, Turkey; Division of Rheumatology, Department of Internal Medicine, Faculty of Medicine, Trakya University, Edirne, Turkey; Division of Rheumatology, Department of Internal Medicine, Faculty of Medicine, Cumhuriyet University, Sivas, Turkey; Clinic of Rheumatology, Department of Internal Medicine, SBU Adana City Training and Research Hospital, Adana, Turkey; Division of Rheumatology, Department of Internal Medicine, Istanbul Provincial Health Directorate, Istanbul Kartal Dr. Lutfi Kırdar Training and Research Hospital, Istanbul, Turkey; Division of Rheumatology, Department of Internal Medicine, Istanbul Provincial Health Directorate, Istanbul Kartal Dr. Lutfi Kırdar Training and Research Hospital, Istanbul, Turkey; Division of Rheumatology, Department of Internal Medicine, Faculty of Medicine, Ege University, Izmir, Turkey; Division of Rheumatology, Department of Internal Medicine, Faculty of Medicine, Hacettepe University, Ankara, Turkey; Division of Rheumatology, Department of Internal Medicine, Faculty of Medicine, Hacettepe University, Ankara, Turkey; Pfizer Turkey, Istanbul, Turkey; Pfizer Turkey, Istanbul, Turkey; Pfizer Turkey, Istanbul, Turkey; Pfizer Turkey, Istanbul, Turkey; Pfizer Turkey, Istanbul, Turkey; Pfizer Turkey, Istanbul, Turkey

**Keywords:** RA, quality of life, patient preference, DAS, ESR, switch, advanced treatment

## Abstract

**Objective:**

We aimed to evaluate quality of life (QoL), disease activity, compliance to treatment, patient and physician preferences for route of administration (RoA), status of health and pain in RA patients starting advanced treatments or needing a switch, and the factors associated with patient preferences.

**Methods:**

A multicentre, prospective, observational and 1-year follow-up study was conducted, between 2015 and 2020, in adult RA patients using advanced treatments for the first time or needing a switch in their current treatments. All the data collected were entered into electronic case report forms. DAS in 28 joints with ESR [DAS28-4(ESR)], EuroQol 5-Dimensional Questionnaire (EQ-5D), HAQ Disability Index (HAQ-DI), Compliance Questionnaire for Rheumatology (CQR-19), Work Productivity and Activity Impairment Instrument (WPAI) and Patient Global Assessment-Visual Analogue Scale (PGA-VAS) questionnaires were used for longitudinal assessments.

**Results:**

Four hundred and fifty-nine patients were enrolled. Three hundred and eight patients (67.1%) attended the final study visit at 12 months and were included for comparative analyses. Irrespective of RoA, the disease activity and QoL improved significantly at 12 months, whereas compliance worsened. At baseline and 12 months, EQ-5D and DAS28-4(ESR) scores were significantly correlated (*P* < 0.001). The WPAI scores changed significantly in favour of better outcomes over 12 months after initiation of advanced treatment or switching (*P* < 0.001). A higher proportion of patients preferred an oral RoA, in comparison to physicians (53.6% *vs* 31.4%; *P* < 0.001). Patient and physician RoA preferences were independent of gender, age, disease duration, advanced treatment type and the EQ-5D-3L, DAS28-4(ESR), HAQ-DI, PGA-VAS and CQR-19 scores at baseline.

**Conclusion:**

The oral route was more frequently preferred by patients compared with physicians, although patients’ preference rates showed a slight increase towards the end of the treatment, which might be an important factor for RA outcomes. Better control of disease activity and QoL were achieved at 12 months, regardless of RoA.

Key messages

- Choices of RA patients and physicians differ for the preferred route of administration of advanced medications.
- Compliance worsens over time, regardless of patient and physician preferences for the route of administration.
- Advanced RA treatments improve quality of life, disease activity, health status and productivity in Turkish patients.

## Introduction

RA is a chronic, systemic, disabling autoimmune disease and the most common form of inflammatory arthritis that causes functional disability, significant pain, joint destruction and premature mortality [1, 2]. The global prevalence of RA is estimated to be 0.24% [3], and prevalence in Turkey was found to be 0.36% [4]. Clinical remission and low disease activity are the main therapeutical goals of RA management. The conventional synthetic (cs) DMARDs continue to be recommended as the first-line treatment of RA, whereas biological (b) and targeted synthetic (ts) DMARDs (advanced RA treatments) are used, with or without csDMARDs, in patients who fail their initial treatment [5, 6]. When remission is not achieved, disease progression is characterized by progressive cartilage and bone damage and pain, leading to significant work disability, deterioration of quality of life (QoL), morbidity and mortality [7–10]. Despite conventional treatments, RA still has many deleterious consequences. From the patients' perspective, these include persistent pain, functional disability, fatigue and depression, modified by health beliefs and underlying psychological problems. Treatment with DMARDs and biologic agents improves pain, fatigue and disability. DMARDs and biologics both significantly reduce HAQ scores, and the reduction is usually maintained for 2–5 years. This improvement is observed in both early and advanced stages of the disease [8]. Frequent assessment of disease activity and response to therapy is crucial for successful long-term management of RA [2].

Given the high disease burden of RA, consideration of patient preferences and medication adherence are crucial for sustained disease control [11]. Patient-reported issues of QoL and disability, however, are consistently reported to be topics of infrequent discussion during clinical follow-up visits [12, 13]. Patient adherence to advanced RA treatments has also been identified as a research question by the EULAR [5].

Assessment of patient preferences in treatment decisions of RA has gained popularity during the last decades [14]. Patient-centred care is thought to cause significant increases in patient satisfaction and treatment adherence levels [15–17]. An increased level of dialogue between RA patients and their physicians also optimizes the management of RA [18, 19]. Previous studies reported variable results regarding patient preferences and choice of medications. Medication efficacy, safety, route of medication administration (RoA), the cost-sharing or the routes for the financial provisions are among the essential factors in this decision-making process [14, 18, 20–23]. The differences associated with geographical place, culture and lifestyle in patient preferences can also lead to significant considerations for the individualized treatment of RA patients [18, 24]. However, overall perceptions of RA patients about their treatment options have been assessed in a limited number of studies [23].

Continuing a previous local study on patient-reported preferences, RoA and unmet needs in advanced RA management [25], we conducted a study to investigate QoL, disease activity, preferences for the RoA, productivity loss, and the compliance of patients treated with advanced RA medications to explore factors related to patient-reported outcomes and the management approach in routine practice.

We hypothesized that patient preferences might be impacted by both patient- and treatment-related factors. In this study, we aimed to examine the association between patient preferences regarding the RoA stratification and the patient-reported clinical outcomes after the use of advanced treatment modalities for RA.

## Methods

### Study design and patients

This study was designed as a 1-year follow-up, national, multicentre, prospective and observational study, which was conducted between August 2015 and January 2020 in 17 study centres. Eligible patients were Turkish citizens ≥18 years of age, with a diagnosis of RA confirmed according to the ACR/EULAR 2010 criteria [26]. Patients could be switching between advanced RA treatments or receiving an advanced RA treatment for the first time at enrolment. Exclusion criteria were cognitive impairment that could prevent study assessments via questionnaires, participation in a clinical trial within the last 4 weeks or within the time frame of five times the half-life of a trial medication, employment with study institutions or the sponsor, and pregnancy or lactation for female subjects. To prevent bias, all available and reimbursed advanced RA treatment options in Turkey [bDMARDs (adalimumab, etanercept, golimumab, infliximab, certolizumab, abatacept, rituximab, and tocilizumab) and tsDMARDs (tofacitinib)] were eligible. The approvals were obtained from Marmara University School of Medicine Clinical Research Ethical Committee (protocol no. A39212770/04.11.2016) and the Ministry of Health before the study commencement, and patients (or their legal representatives, if needed) signed informed consent (20.09.2016/V3) for study participation. A decision to initiate or change advanced RA treatment was not taken solely for the inclusion of participants in this study. The observational nature of the study in a real-world setting was protected.

### Objectives and assessments

The primary objective of the study was to evaluate QoL and disease activity using DAS in 28 joints with ESR [DAS28-4(ESR)] over a 1-year period after initiation of advanced RA treatment or switching. Secondary objectives were to determine general health, disease status, pain status, productivity loss, patient compliance, and patient and physician preferences for the route of drug administration (oral or parenteral), along with prespecified interrelationships. In the scope of this study, investigators obtained the patient data from electronic or written patient records of the participating centres and entered these data on electronic case report forms. For some questionnaires, the data required were obtained directly from the patients via patient interviews and from medical records.

Data of the patients were archived in various ways in the study centres. One of the most commonly used data sources was the computer-based patient recording system.

Patient preferences were determined through a questionnaire, and physician preferences were determined according to the choice of treatment applied to the patient. Physicians decide their treatments considering patients’ general health status, socioeconomic and sociodemographic status and living conditions (urban/rural). Patients were evaluated at baseline (before the advanced RA therapy initiation or switch) and at 12 months, using the EuroQol Five-Dimensional Questionnaire, 3-level version (EQ-5D-3L; [27, 28]), the HAQ Disability Index (HAQ-DI; [29, 30]), DAS in 28 joints with ESR rate (DAS28-4[ESR]; [31, 32]), Compliance Questionnaire for Rheumatology (CQR-19; [33, 34]), Patient Global Assessment on a 100-mm visual analogue scale (PGA-VAS; [35]), Work Productivity and Activity Impairment Instrument (WPAI; [36–38]) and some additional questions, such as employment status, salary range, working hours per week for employed patients, loss of productive workdays within the last 3 months attributable to RA, number of days spent at home within the last 3 months attributable to RA flare, loss of productive workdays within the last 3 months attributable to RA flare requiring a hospital visit, loss of productive workdays within the last 3 months attributable to RA treatment administration, and duration of unemployment attributable to RA.

Higher scores indicate worse outcomes for HAQ-DI, DAS28-4(ESR) and WPAI and related questions, and better outcomes for EQ-5D-3L, PGA-VAS and CQR-19. Across all EQ-5D dimensions (mobility, self-care, usual activities, pain/discomfort and anxiety/depression), the levels recognize ‘no problems, slight problems, moderate problems, severe problems, and unable to do/extreme’. Each dimension in the EQ-5D-5L has five response levels: no problems (level 1), slight problems (level 2), moderate problems (level 3), severe problems (level 4) and extreme problems (level 5). For CQR-19 findings, unsatisfactory compliance was defined as a score of ≤80% [39]. The DAS28-4(ESR) score includes both bDMARDS initiated and switched patients.

### Statistical analysis

A statistical calculation projected that 697 patients should be included in the study, with 95% power and ±3% accuracy to detect a significant relative increase of patients with low disease activity [estimated that 19% of patients at 6 months would present with a DAS28-4(ESR) score of <3.2]. Descriptive statistics are presented as the mean and s.d. or median (range) based on the distribution of data. Categorical variables are expressed as numbers and percentages. Normality was assessed with the Shapiro–Wilk test. Patients who completed questionnaires both at baseline and at 12 months were included in the analyses to assess change in questionnaire findings over time. The Wilcoxon signed rank and McNemar tests were used for repeated non-parametric comparisons (i.e. questionnaire scores and RoA preferences, respectively). Student’s paired *t* test was used to compare baseline and 12-month ESR and CRP levels. The Mann–Whitney *U* test was used to compare patient preferences and physician prescriptions. To explore relationships between QoL and disease activity or compliance, correlation analyses were carried out for “EQ-5D-3L and DAS28-4(ESR)” and for “EQ-5D-3L and CQR-19” findings at baseline and 12 months, using Spearman's rank correlation.

Binary logistic regression analysis was carried out to evaluate the effects of demographic and baseline disease characteristics on the preferred or prescribed administration routes at baseline. RoA was selected as the dependent variable, and age, gender, duration of RA, previous advanced RA treatment exposure, EQ-5D-3L, HAQ-DI, DAS28-4(ESR), PGA-VAS and CQR-19 were selected as independent variables. For statistical analysis, Jamovi (v.1.0.8, retrieved from https://www.jamovi.org/) was used. In statistical analysis, the significance level (*P*-value) was considered as a two-sided value of 0.05.

## Results

In total, 470 patients were evaluated. Eleven patients were excluded from the analysis set owing to violation of eligibility criteria (diagnoses other than RA), and 459 patients with moderately to severely active RA receiving advanced treatments were enrolled for the final analysis. Of these, 308 patients (67.1%) attended the study visit at 12 months. Patient demographics and baseline disease characteristics are shown in Table 1.

**Table 1.** Patient demographics, clinical characteristics and treatments

Characteristic	Enrolment (*n* = 459)	
Age, mean (s.d.), years	50.2 (12.0)	
Female, *n* (%)	349 (76.0)	
Education level, *n* (%)		
Illiterate	38 (8.3)	
Primary school graduate	219 (47.7)	
Secondary school graduate	49 (10.7)	
High-school graduate	90 (19.6)	
University graduate	59 (12.8)	
Postgraduate	4 (0.9)	
Employed, *n* (%)	108 (23.5)	
Duration of RA, mean (s.d.), years^a^	10.1 (7.9)	
Previous advanced RA treatment, *n* (%)		
No	358 (78.0)	
Yes	101 (22.0)	
**RA treatments, *n* (%)**	**Enrolment (*n* = 459)**	**12 months (*n* = 308)**
tsDMARD		
Tofacitinib (p.o. 5 mg twice a day)	143 (31.2)	93 (30.2)
bDMARD		
TNFi		
Adalimumab (s.c. 40 mg every 2 weeks)	46 (10.0)	28 (9.1)
Etanercept (s.c. 25 mg twice a week)	40 (8.7)	35 (11.4)
Golimumab (s.c. 50 mg every 4 weeks)	21 (4.6)	12 (3.9)
Infliximab (i.v. 3mg/kg 0, 2, 6 weeks)	8 (1.7)	5 (1.6)
Certolizumab (s.c. 200 or 400** **mg every 2 weeks)	44 (9.6)	30 (9.7)
Non-TNFi		
Abatacept (i.v. 500, 750 or 1000** **mg 0, 2, 4** **weeks)	40 (8.7)	23 (7.5)
Rituximab (i.v. 1000** **mg in 2 doses every 24** **weeks)	69 (15.0)	53 (17.2)
Tocilizumab (i.v. 8** **mg/kg every 4** **weeks)	48 (10.5)	29 (9.4)
ESR, mean (s.d.), mm/h^b^	35.0 (22.2)	23.6 (19.2)
CRP, mean (s.d.), mg/l^b^	20.3 (25.3)	8.8 (12.9)

a For duration of RA, *n* = 458.

b *P* < 0.001 for baseline *vs* 12-month levels.

bDMARD: biologic DMARD; CQR-19: Compliance Questionnaire for Rheumatology; csDMARD: conventional synthetic DMARD; DAS28-4(ESR): DAS in 28 joints with ESR; HAQ-DI: HAQ-Disability Index; TNFi: TNF inhibitor; tsDMARD: targeted synthetic DMARD.

The majority of patients (76.0%) were female, and their mean age was 50.2 (12.0) years. At baseline, 351 patients (76.5%) were unemployed. Of the unemployed patients, 238 (67.8%) stated being a housewife and 65 (18.5%) stated being retired. The mean duration of RA was 10.1 (7.9) years. HCQ, MTX and LEF were the most commonly prescribed medications for RA before study enrolment (48.2, 47.1 and 42.7%, respectively). At the beginning of the study, prescribed medications and the number of patients receiving them were as follows: tofacitinib 143 (31.2%); rituximab 69 (15.0%); tocilizumab 48 (10.5%); adalimumab 46 (10.0%); certolizumab pegol 44 (9.6%); etanercept 40 (8.7%); abatacept 40 (8.7%); golimumab 21 (4.6%); and infliximab 8 (1.7%). In addition to prescribed medications at the beginning of the study, the active medications used at 3, 6, 9 and 12 months of the study are given in Supplementary Table S1, available at *Rheumatology Advances in Practice* online. Advanced RA treatment was initiated in 358 patients (78.0%) who were previously receiving csDMARDs, whereas medication was changed in 101 (22.0%) patients who were previously treated with an advanced RA treatment. During the follow-up, advanced RA treatments were changed in 57 patients (12.0%) (Supplementary Table S2, available at *Rheumatology Advances in Practice* online). No patients died during the study period.

EQ-5D-3L and DAS28-4(ESR) scores changed significantly in favour of better outcomes over 12 months after advanced treatment initiation or switching (*P* < 0.001 for both; Table 2; Supplementary Tables S3 and S4, available at *Rheumatology Advances in Practice* online).

**Table 2.** Questionnaire scores at baseline and 12 months

Questionnaire	Baseline (*n* = 308)	12 months (*n* = 308)	*P*-value
EQ-5D-3L	0.52 (−0.59 to +1.00)	0.73 (−0.24 to +1.00)	<0.001
DAS28-4(ESR)	5.05 (1.41–8.33)	2.84 (0–7.26)	<0.001
HAQ-DI	0.90 (0–2.90)	0.30 (0–2.50)	<0.001
CQR-19	73.68 (17.5–96.5)	68.42 (5.26–89.5)	<0.001
PGA-VAS	60.0 (0–100)	30.0 (0–100)	<0.001
WPAI^a^ (%)			
Absenteeism	0 (0–100)	0 (0–100)	0.062
Presenteeism	50 (0–100)	20 (10–90)	<0.001
Overall work impairment	60 (10–100)	20 (10–100)	<0.001
Activity impairment	50 (10–100)	20 (10–90)	<0.001

Values are shown as the median (range).

a *n* = 63 for WPAI completers.

*P* < 0.001 for baseline *vs* 12-month levels.

CQR-19: Compliance Questionnaire for Rheumatology; DAS28-4(ESR): DAS in 28 joints with ESR; EQ-5D-3L: EuroQol Five-Dimensional Questionnaire 3-level version; HAQ-DI: HAQ-Disability Index; PGA-VAS: Patient Global Assessment on a 100-mm Visual Analogue Scale; WPAI: Work Productivity and Activity Impairment Instrument.

At study initiation, 246 patients (53.6%) preferred an oral route for the administration of advanced treatments, whereas 213 (46.4%) preferred a parenteral route. Patient preferences for the RoA were similar at 12 months (50% preferred oral or parenteral route; *P* > 0.05; Supplementary Table S5, available at *Rheumatology Advances in Practice* online). However, there was a significant difference between baseline patient preferences and physician prescriptions regarding the RoA, in that physicians favoured an advanced RA medication administered via a parenteral route more frequently (46.4% preferred oral route *vs* 68.6% preferred a parenteral route, *P* < 0.001). In 38.3% of cases, physicians prescribed advanced treatments with routes of administration that differed from patient preferences. Patient preferences for the RoA between advanced treatment-naïve and treatment-experienced groups were similar at baseline (48.5% *vs* 55.0% for oral route, *P* = 0.361). When patients were classified according to administration routes of advanced therapies, baseline and 12-month questionnaire scores were similar for oral and parenteral route groups (*P* > 0.05 for all questionnaires; Table 3).

**Table 3.** Questionnaire results at baseline and 12 months according to route of administration

Questionnaire	All patients,a median (min–max) (*n* = 308)	Oral route, median (min–max) (*n* = 94)	Parenteral route, median (min–max) (*n* = 214)	*P*-value (oral *vs* parenteral)
EQ-5D-3L				
Baseline	0.52 (0.59–1.00)	0.52 (0.24–1.0)	0.52 (0.59–1)	0.698
12** **months	0.73 (0.24–1.00)	0.72 (0.18–1.00)	0.73 (0.24–1.00)	0.962
DAS28-4(ESR)				
Baseline	5.05 (1.41–8.33)	5.02 (2.09–7.83)	5.05 (1.41–8.33)	0.831
12** **months	2.84 (0–7.26)	2.96 (0–7.26)	2.81 (0.28–7.26)	0.159
HAQ-DI				
Baseline	0.90 (0–2.90)	0.90 (0–2.70)	0.95 (0–2.90)	0.384
12** **months	0.30 (0–2.50)	0.30 (0–2.35)	0.30 (0–2.50)	0.649
PGA-VAS				
Baseline	60.0 (0–100)	70.0 (5–100)	60.0 (0–100)	0.543
12** **months	30.0 (0–100)	25.0 (0–80)	30.0 (0–100)	0.903
WPAI-Absenteeism (work time missed)^b^				
Baseline	0 (0–100)	0 (0–40)	0 (0–100)	0.519
12** **months	0 (0–100)	0 (0–55)	0 (0–100)	0.846
WPAI-Presenteeism (impairment at work)^b^				
Baseline	50 (0–100)	60 (10–90)	50 (0–100)	0.211
12** **months	20 (10–90)	20 (10–80)	20 (10–90)	0.725
WPAI-Overall work impairment^b^				
Baseline	60 (10–100)	60 (10–90)	60 (10–100)	0.795
12** **months	20 (10–100)	20 (10–87)	20 (10–100)	0.684
WPAI-Activity impairment^b^				
Baselines	50 (10–100)	50 (10–100)	50 (10–100)	0.331
12** **months	20 (10–90)	20 (10–80)	20 (10–90)	0.916

a For EQ-5D, DAS28-4(ESR), HAQ-DI, CQR-19 and PGA-VAS scores and WPAI percentages (except absenteeism), *P* < 0.001 for all patients from baseline to 12 months.

b For WPAI percentages, *n* = 63 for all, *n* = 22 for oral route and *n* = 41 for parenteral route.

CQR-19: Compliance Questionnaire for Rheumatology; DAS28-4(ESR): DAS in 28 joints with ESR; EQ-5D-3L: EuroQol Five-Dimensional Questionnaire 3-level version; HAQ-DI: HAQ-Disability Index; PGA-VAS: Patient Global Assessment on a 100-mm Visual Analogue Scale; WPAI: Work Productivity and Activity Impairment Instrument.

**Figure 1.**
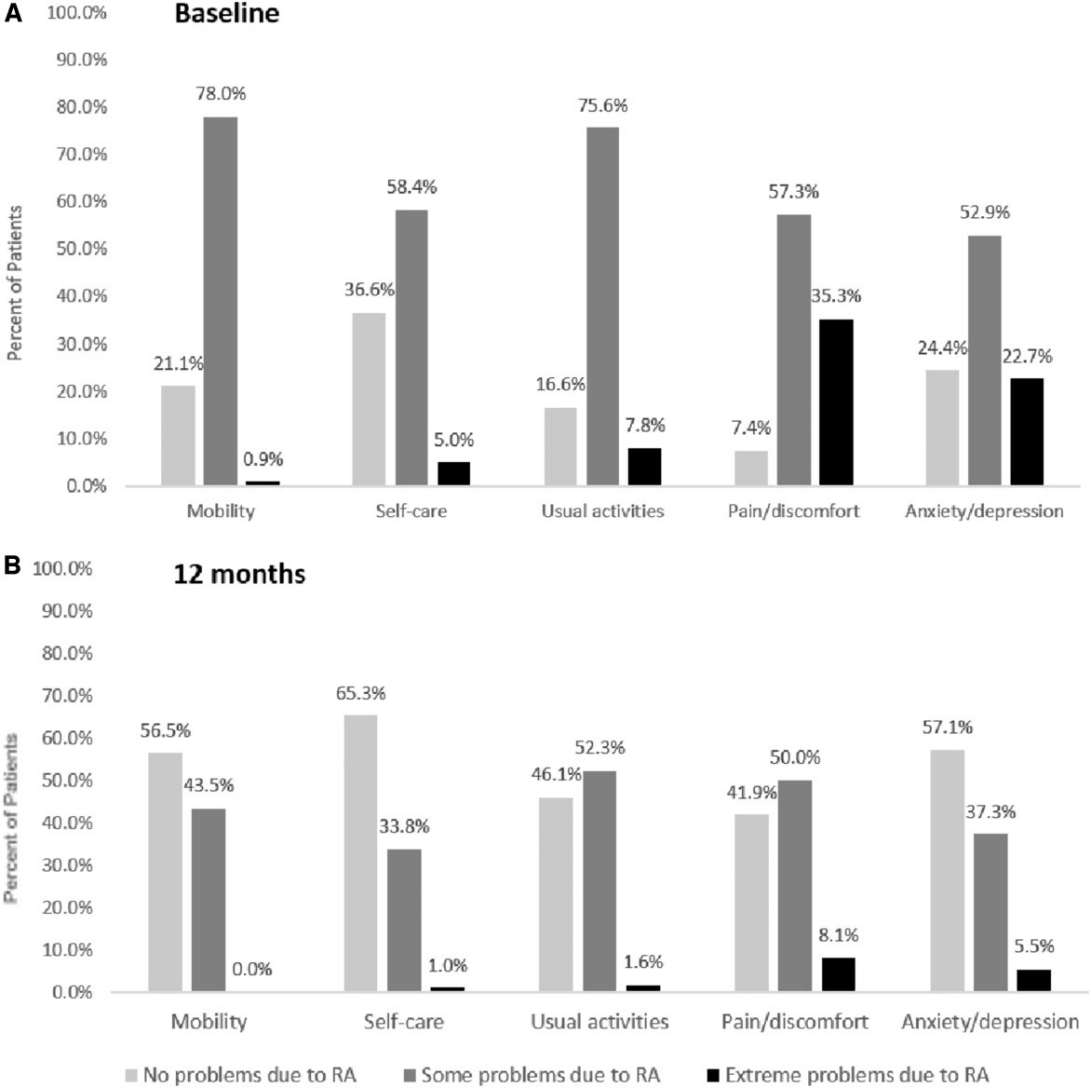
EuroQol 5-Dimensional Questionnaire patient responses per domain. EQ-5D-3L patient responses at **(A)** baseline (*n* = 459) and **(B)** 12 months (*n* = 308). EQ-5D-3L: EuroQol 5-Dimensional Questionnaire

**Figure 2.**
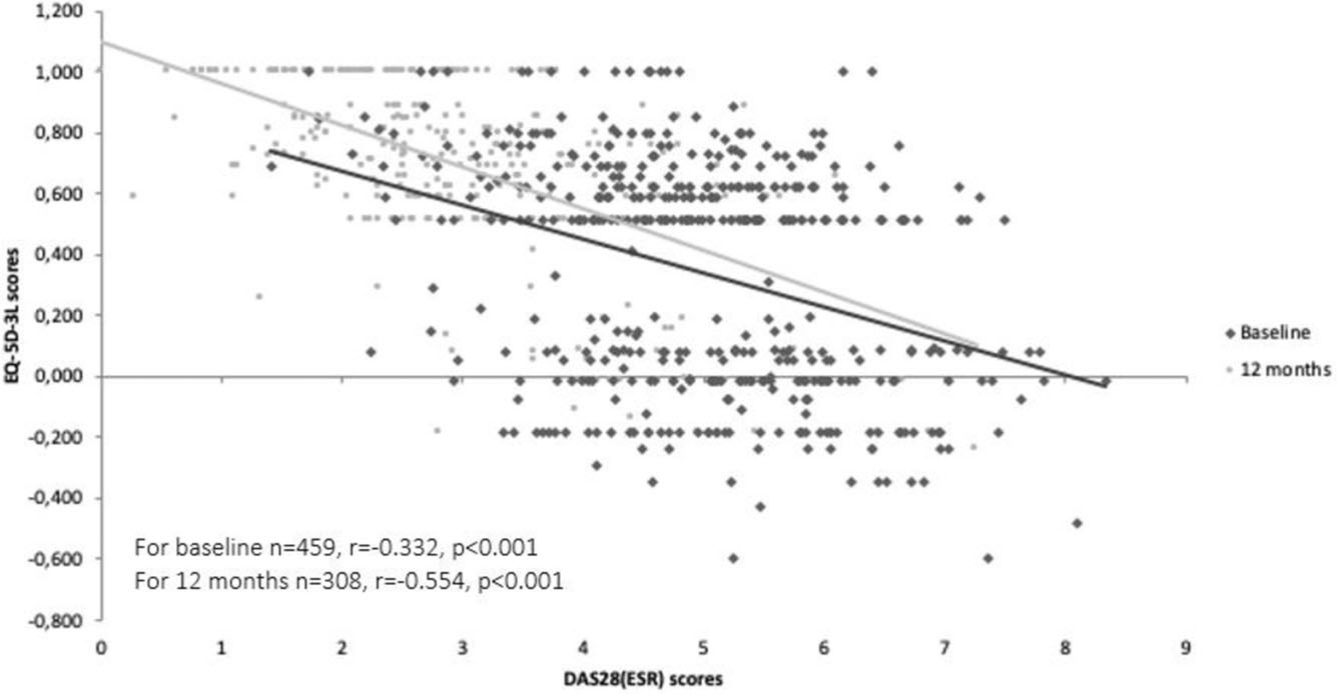
Correlation curve between EuroQol 5-Dimensional Questionnaire and DAS in 28 joints with ESR scores at baseline and at 12 months. EQ-5D-3L: EuroQol 5-Dimensional Questionnaire; DAS28-4(ESR): DAS in 28 joints with ESR

At baseline and 12 months, 35.3 and 8.1% of patients, respectively, reported extreme problems attributable to RA in the pain/discomfort domain of EQ-5D-3L, and 22.7 and 5.5% of patients, respectively reported extreme problems attributable to RA in the anxiety/depression domain. The proportions of patients indicating no problems related to RA increased by 25% or more within all EQ-5D-3L domains (Fig. 1; Supplementary Fig. S1, available at *Rheumatology Advances in Practice* online). A significant negative correlation was found between EQ-5D-3L and DAS28-4(ESR) scores at baseline (*n* = 459, *r* = −0.332, *P* < 0.001) and at 12 months (*n* = 308, *r* = −0.554, *P* < 0.001; Fig. 2).

For the RoA, both patient preferences and physician prescriptions were found to be independent of patient gender and age, duration of RA, previous exposure to advanced RA treatment, EQ-5D-3L, DAS2-4(ESR), HAQ-DI, PGA-VAS and CQR-19 scores at baseline (Table 4).

**Table 4.** Binary logistic regression analyses of factors affecting the preference of patients and physicians for administration routes at baseline

Independent variables	*P*-value for preference	
	Patients (*n* = 459)	Physicians (*n* = 459)
Gender	0.605	0.225
Age	0.804	0.342
Duration of RA	0.379	0.075
Previous advanced RA treatment exposure	0.247	0.602
DAS28-4(ESR)	0.058	0.822
HAQ-DI	0.672	0.070
PGA-VAS	0.052	0.546
EQ-5D-3L	0.354	0.735
CQR-19^a^	0.204	0.583

a Data were collected before prescription.

CQR-19: Compliance Questionnaire for Rheumatology; DAS28-4(ESR): DAS in 28 joints with ESR; EQ-5D-3L: EuroQol 5-Dimensional Questionnaire; HAQ-DI: HAQ Disability Index; PGA-VAS: Patient Global Assessment-Visual Analogue Scale.

WPAI (except for absenteeism domain) scores changed significantly in favour of better outcomes over 12 months after advanced treatment initiation or switching (*P* < 0.001; Table 2). HAQ-DI and PGA-VAS scores also changed significantly in favour of better outcomes over 12 months after advanced treatment initiation or switching (*P* < 0.001 for both; Table 2).

CQR-19 scores decreased (73.7% at baseline vs. 68.4% at 12 months, *P* < 0.001; Table 2) and the proportion of patients with unsatisfactory compliance increased slightly. Significant correlations were also found at 12 months between CQR-19 and EQ-5D-3L (*n* = 308, *r* = 0.134, *P* = 0.019) and CQR-19 and DAS28-4(ESR) scores (*n* = 308, *r* = 0.187, *P* < 0.001) but not at baseline.

Although an initial sample size of 697 patients was estimated to be needed, we were able to enrol 470 patients throughout the study period. However, in a post-study sensitivity analysis, it was observed that 56.2% of patients had a DAS28-4(ESR) score of <3.2 at 12 months. The precision of the study was calculated to be 5.5% at a significance level of α = 0.05. The analysis of additional questions was not found to be statistically significant (*P* > 0.05).

## Discussion

In this longitudinal observational study, we observed that the oral route was more frequently preferred at the initiation of the advanced RA treatment, with a slight difference among the patients regarding the parenteral route. However, this difference was lost at the end of 1 year. Contrary to the patients, the parenteral route was more frequently prescribed by the physicians, with a discordance rate of 38.3% at the initiation of the treatment. We also showed that the advanced RA treatment initiation or switching was associated with better QoL, general health status and disease activity over 12 months, with >50% of the patients reporting a low disease activity. These findings are in line with previous reports of improved patient-reported outcomes with use of advanced RA treatments [40, 41]. Likewise, a previous local study indicated better disease control and health status in RA patients who were on biological therapy when compared with patients who were eligible for biological therapy but not receiving it [25]. A weak to moderate negative correlation was found between EQ-5D-3L and DAS28-4(ESR) scores at baseline and 12 months, which is also similar to earlier reports [42, 43].

In our study, about half of the patients preferred an oral route for the administration of RA therapies at baseline and 12 months. Similar findings were reported by Louder *et al.* [14] in a simulated analysis and by Alten *et al.* [17] in a discrete choice experiment, reporting that 56 and 49% of patients, respectively, prefer an oral route for RA treatment. An international study also reported that 57% of RA patients preferred an oral route for their treatment. Speed and ease of administration were the most commonly stated reasons for the preference of an oral route [18]. In contrast, the preferences of physicians were somewhat different from those of the patients; in other words, we found a discrepancy between patient preferences and physician prescriptions regarding the RoA of advanced RA medication initiation or switching. More than two-thirds of the rheumatologists prescribed an advanced RA therapy to be administered via a parenteral route. The reason for the preference of a parenteral route by physicians might be a general belief in medical practice, stated as ‘The evident advantages of parenteral injection are the reliability and precision of dosage and the generally rapid onset of action of the drug’ [44] or a habit obtained during MTX prescription, because of its higher bioavailability and compliance in parenteral administration [45]. We did not identify any disease- or outcome-related factor associated with patient or physician choice for the RoA. Although evidence is lacking in the scientific literature, meeting patient preferences and shared decision-making between patients and physicians were estimated or expected to improve medication compliance [17, 18].

Medication compliance in RA has been evaluated in several studies with broadly varied methodology, using subjective reporting, validated surveys or drug monitoring to measure adherence. Validated survey results indicate compliance rates of 50–75% [46, 47], whereas stricter criteria point to compliance rates of 30–40% [48–50]. This finding was observed consistently, irrespective of the RoA. Although improved QoL and disease activity were associated with worse compliance at 12 months, the strength of correlations was very weak. The rate of non-compliance to advanced RA treatments has been reported to be 40% immediately after prescription of medication, and this rate did not differ across RoA and drugs [51]. Decreased medication compliance in inflammatory arthritis was also reported over a period of 6–12 months [52]. Observational studies examining compliance in RA have involved different drugs, patient groups and compliance measures, and differing results for factors associated with non-compliance have been published [46, 53]. These studies, however, consistently indicate a suboptimal compliance rate in RA patients, as our findings do, and some novel approaches have been suggested to improve patient education and compliance [53, 54]. However, the severity of RA is also proposed as a factor for medication adherence, and some patients might show non-adherent behaviour because they feel better after a period of the treatment [55].

### Limitations

Although we failed to meet the prespecified sample size, the effect size-based proportion of patients with low disease activity [DAS28-4(ESR) score <3.2] turned out to be much higher than expected, and the margin of error within our study was found to be acceptable with the number of enrolled patients. The WPAI questionnaire and questions related to productivity were completed by a relatively low number of patients, because the employment ratio was low in our study cohort. Additionally, to help improve patient care, literacy might be a limitation for this study. We did not seek specifically to examine the relationship between patient and physician preferences for the RoA and medication compliance. This aspect, however, could be the objective of future studies considering the difference between the approaches of patients and health-care professionals for the RoA. The discordance between patient and physician preferences should be evaluated further in future studies.

### Conclusions

We observed that the choices of patients and physicians differed for the preferred RoA of advanced medications. The physicians more frequently preferred parenteral routes. The preference rate for the oral route by patients showed a slight decrease towards the end of 1 year. The initiation or switching of advanced RA treatments was associated with better QoL, control of disease activity, productivity and health status at 12 months. Compliance, however, worsened over time, regardless of the RoA. Dedicated future studies investigating the approach of patients and physicians for the preferred RoA of advanced RA medication might potentially contribute to management strategies for improved patient compliance.

## Supplementary data

Supplementary data are available at Rheumatology Advances in Practice online.

## Supplementary Material

rkac071_Supplementary_Data

## Data Availability

The authors confirm that the data supporting the findings of this study are available within the article and its supplementary materials.

## References

[1] Alamanos Y , Drosos AA. Epidemiology of adult rheumatoid arthritis. Autoimmun Rev 2005;4:130–6. 15823498 10.1016/j.autrev.2004.09.002

[2] Lee DM , Weinblatt ME. Rheumatoid arthritis. Lancet 2001;358:903–11. 11567728 10.1016/S0140-6736(01)06075-5

[3] Cross M , Smith E, Hoy D et al The global burden of rheumatoid arthritis: estimates from the global burden of disease 2010 study. Ann Rheum Dis 2014;73:1316–22. 24550173 10.1136/annrheumdis-2013-204627

[4] Akkoc N , Akar S. Epidemiology of rheumatoid arthritis in Turkey. Clin Rheumatol 2006;25:560–1. 16397748 10.1007/s10067-005-0092-2

[5] Smolen JS , Landewé RBM, Bijlsma JWJ et al EULAR recommendations for the management of rheumatoid arthritis with synthetic and biological disease-modifying antirheumatic drugs: 2019 update. Ann Rheum Dis 2020;79:685–99. 31969328 10.1136/annrheumdis-2019-216655

[6] Singh JA , Saag KG, Bridges SL Jr et al American College of Rheumatology Guideline for the treatment of rheumatoid arthritis. Arthritis Rheumatol 2016;68:1–26. 10.1002/art.39480 26545940

[7] Pincus T , Brooks RH, Callahan LF. Prediction of long-term mortality in patients with rheumatoid arthritis according to simple questionnaire and joint count measures. Ann Intern Med 1994;120:26–34. 8250453 10.7326/0003-4819-120-1-199401010-00005

[8] Pollard L , Choy EH, Scott DL. The consequences of rheumatoid arthritis: quality of life measures in the individual patient. Clin Exp Rheumatol 2005;23:S43–52. 16273784

[9] Solomon DH , Karlson EW, Rimm EB et al Cardiovascular morbidity and mortality in women diagnosed with rheumatoid arthritis. Circulation 2003;107:1303–7. 12628952 10.1161/01.cir.0000054612.26458.b2

[10] Sokka T , Kautiainen H, Pincus T et al; the QUEST-RA study group. Work disability remains a major problem in rheumatoid arthritis in the 2000s: data from 32 countries in the QUEST-RA study. Arthritis Res Ther 2010;12:R42. 20226018 10.1186/ar2951 PMC2888189

[11] Thompson A. Practical aspects of therapeutic intervention in rheumatoid arthritis. J Rheumatol Suppl 2009;82:39–41. 19509329 10.3899/jrheum.090130

[12] Rupp I , Boshuizen HC, Dinant HJ, Jacobi CE, van den Bos GA. Disability and health-related quality of life among patients with rheumatoid arthritis: association with radiographic joint damage, disease activity, pain, and depressive symptoms. Scand J Rheumatol 2006;35:175–81. 16766363 10.1080/03009740500343260

[13] McInnes IB , Combe B, Burmester G. Understanding the patient perspective – results of the Rheumatoid Arthritis: Insights, Strategies & Expectations (RAISE) patient needs survey. Clin Exp Rheumatol 2013;31:350–7. 23406685

[14] Louder AM , Singh A, Saverno K et al Patient preferences regarding rheumatoid arthritis therapies: a conjoint analysis. Am Health Drug Benefits 2016;9:84–93. PMC4856233 27182427

[15] Kjeken I , Dagfinrud H, Mowinckel P et al Rheumatology care: involvement in medical decisions, received information, satisfaction with care, and unmet health care needs in patients with rheumatoid arthritis and ankylosing spondylitis. Arthritis Rheum 2006;55:394–401. 16739186 10.1002/art.21985

[16] Barton JL. Patient preferences and satisfaction in the treatment of rheumatoid arthritis with biologic therapy. Patient Prefer Adherence 2009;3:335–44. 20016797 10.2147/ppa.s5835 PMC2792871

[17] Alten R , Krüger K, Rellecke J et al Examining patient preferences in the treatment of rheumatoid arthritis using a discrete-choice approach. Patient Prefer Adherence 2016;10:2217–28. 27843301 10.2147/PPA.S117774 PMC5098563

[18] Taylor PC , Betteridge N, Brown TM et al Treatment mode preferences in rheumatoid arthritis: moving toward shared decision-making. Patient Prefer Adherence 2020;14:119–31. 32021123 10.2147/PPA.S220714 PMC6980841

[19] Gibofsky A , Galloway J, Kekow J et al; RA NarRAtive global advisory panel. Comparison of patient and physician perspectives in the management of rheumatoid arthritis: results from global physician- and patient-based surveys. Health Qual Life Outcomes 2018;16:211. 30413162 10.1186/s12955-018-1035-3 PMC6230272

[20] Bridges JFP , Hauber AB, Marshall D et al Conjoint analysis applications in health—a checklist: a report of the ISPOR Good Research Practices for Conjoint Analysis Task Force. Value Health 2011;14:403–13. 21669364 10.1016/j.jval.2010.11.013

[21] Fraenkel L , Bogardus ST, Concato J et al Patient preferences for treatment of rheumatoid arthritis. Ann Rheum Dis 2004;63:1372–8. 15020312 10.1136/ard.2003.019422 PMC1754807

[22] Zhu B , Chang L, Qian L et al RA medication preferences among U.S. patients in an online rheumatoid arthritis community. Arthritis Rheumatol 2016;68(Suppl 10):2238.

[23] Choi IA , Kim JH, Hae Chang S et al Patient perspectives on biological treatments for inflammatory arthritis: a multi-center study in Korea. Arch Rheumatol 2021;36:499–509. 35382362 10.46497/ArchRheumatol.2021.8524 PMC8957778

[24] Kumar K , Klocke R. Ethnicity in rheumatic disease. Clin Med (Lond) 2010;10:370–2. 20849012 10.7861/clinmedicine.10-4-370 PMC4952167

[25] Direskeneli H , Akkoç N, Bes C et al Impact of rheumatoid arthritis in Turkey: a questionnaire study. Clin Exp Rheumatol 2014;32:477–83. 24960289

[26] Aletaha D , Neogi T, Silman AJ et al 2010 Rheumatoid arthritis classification criteria: an American College of Rheumatology/European League Against Rheumatism collaborative initiative. Arthritis Rheum 2010;62:2569–81. 20872595 10.1002/art.27584

[27] Rabin R , de Charro F. EQ-5D: a measure of health status from the EuroQol Group. Ann Med 2001;33:337–43. 11491192 10.3109/07853890109002087

[28] Kaya N , Babadag K. Health related quality of life in patients with rheumatoid arthritis. Istanbul University FNHYO 2004;13:51–71.

[29] Ramey DR , Raynauld JP, Fries JF. The health assessment questionnaire 1992: status and review. Arthritis Care Res 1992;5:119–29. 1457486 10.1002/art.1790050303

[30] Küçükdeveci AA , Sahin H, Ataman S, Griffiths B, Tennant A. Issues in cross-cultural validity: example from the adaptation, reliability, and validity testing of a Turkish version of the Stanford Health Assessment Questionnaire. Arthritis Rheum 2004;51:14–9. 14872450 10.1002/art.20091

[31] Fuchs HA , Brooks RH, Callahan LF, Pincus T. A simplified twenty-eight-joint quantitative articular index in rheumatoid arthritis. Arthritis Rheum 1989;32:531–7. 2719728 10.1002/anr.1780320504

[32] Sunar I , Yilmaz Tasdelen O, Garip Cimen Y, Bodur H. Translation and validation of the Turkish language version of the Rheumatoid Arthritis Disease Activity Index-5. Int J Rheum Dis 2017;20:2012–9. 24725498 10.1111/1756-185X.12371

[33] de Klerk E , van der Heijde D, van der Tempel H, van der Linden S. Development of a questionnaire to investigate patient compliance with antirheumatic drug therapy. J Rheumatol 1999;26:2635–41. 10606375

[34] Cinar FI , Cinar M, Yilmaz S et al Cross-cultural adaptation, reliability, and validity of the Turkish Version of the Compliance Questionnaire on Rheumatology in patients with Behçet's disease. J Transcult Nurs 2016;27:480–6. 25801762 10.1177/1043659615577699

[35] Challa DNV , Crowson CS, Davis JM. The patient global assessment of disease activity in rheumatoid arthritis: identification of underlying latent factors. Rheumatol Ther 2017;4:201–8. 28488206 10.1007/s40744-017-0063-5 PMC5443732

[36] Reilly MC , Zbrozek AS, Dukes EM. The validity and reproducibility of a work productivity and activity impairment instrument. Pharmacoeconomics 1993;4:353–65. 10146874 10.2165/00019053-199304050-00006

[37] Zhang W , Bansback N, Boonen A et al Validity of the work productivity and activity impairment questionnaire - general health version in patients with rheumatoid arthritis. Arthritis Res Ther 2010;12:R177. 20860837 10.1186/ar3141 PMC2991008

[38] Reilly Associates. WPAI Translations. http://www.reillyassociates.net/WPAI_Translations-2.html (May 2014, date last accessed).

[39] de Klerk E , van der Heijde D, Landewé R, van der Tempel H, van der Linden S. The compliance-questionnaire-rheumatology compared with electronic medication event monitoring: a validation study. J Rheumatol 2003;30:2469–75. 14677194

[40] Inotai A , Rojkovich B, Fülöp A et al Health-related quality of life and utility in patients receiving biological and non-biological treatments in rheumatoid arthritis. Rheumatol Int 2012;32:963–9. 21243499 10.1007/s00296-010-1721-x

[41] Strand V , Lee EB, Yazici Y et al Evaluation of disease activity in patients with rheumatoid arthritis treated with tofacitinib by RAPID3: Post hoc analyses from two phase 3 trials. Clin Rheumatol 2018;37:2043–53. 29656373 10.1007/s10067-018-4077-3 PMC6061070

[42] Hurst NP , Kind P, Ruta D, Hunter M, Stubbings A. Measuring health-related quality of life in rheumatoid arthritis: validity, responsiveness and reliability of EuroQol (EQ-5D). Br J Rheumatol 1997;36:551–9. 9189057 10.1093/rheumatology/36.5.551

[43] Ariza-Ariza R , Hernández-Cruz B, Carmona L et al; Costs and Quality of Life in Rheumatoid Arthritis Study Group. Assessing utility values in rheumatoid arthritis: a comparison between time trade-off and the EuroQol. Arthritis Rheum 2006;55:751–6. 17013822 10.1002/art.22226

[44] Ballard BE. Biopharmaceutical considerations in subcutaneous and intramuscular drug administration. J Pharm Sci 1968;57:357–78. 4871917 10.1002/jps.2600570301

[45] Cipriani P , Ruscitti P, Carubbi F, Liakouli V, Giacomelli R. Methotrexate in rheumatoid arthritis: optimizing therapy among different formulations. Current and emerging paradigms. Clin Ther 2014;36:427–35. 24612941 10.1016/j.clinthera.2014.01.014

[46] van den Bemt BJ , Zwikker HE, van den Ende CH. Medication adherence in patients with rheumatoid arthritis: a critical appraisal of the existing literature. Expert Rev Clin Immunol 2012;8:337–51. 22607180 10.1586/eci.12.23

[47] Pasma A , van't Spijker A, Hazes JM, Busschbach JJ, Luime JJ. Factors associated with adherence to pharmaceutical treatment for rheumatoid arthritis patients: a systematic review. Semin Arthritis Rheum 2013;43:18–28. 23352247 10.1016/j.semarthrit.2012.12.001

[48] Park DC , Hertzog C, Leventhal H et al Medication adherence in rheumatoid arthritis patients: older is wiser. J Am Geriatr Soc 1999;47:172–83. 9988288 10.1111/j.1532-5415.1999.tb04575.x

[49] Viller F , Guillemin F, Briançon S et al Compliance to drug treatment of patients with rheumatoid arthritis: a 3 year longitudinal study. J Rheumatol 1999;26:2114–22. 10529126

[50] Tuncay R , Eksioglu E, Cakir B, Gurcay E, Cakci A. Factors affecting drug treatment compliance in patients with rheumatoid arthritis. Rheumatol Int 2007;27:743–6. 17216476 10.1007/s00296-006-0299-9

[51] Kan HJ , Dyagilev K, Schulam P et al Factors associated with physicians’ prescriptions for rheumatoid arthritis drugs not filled by patients. Arthritis Res Ther 2018;20:79. 29720237 10.1186/s13075-018-1580-5 PMC5932861

[52] de Klerk E , van der Heijde D, Landewé R et al Patient compliance in rheumatoid arthritis, polymyalgia rheumatica, and gout. J Rheumatol 2003;30:44–54. 12508389

[53] Marengo MF , Suarez-Almazor ME. Improving treatment adherence in patients with rheumatoid arthritis: what are the options? Int J Clin Rheumtol 2015;10:345–56. 27087857 10.2217/ijr.15.39 PMC4826730

[54] Joplin S , van der Zwan R, Joshua F, Wong PKK. Medication adherence in patients with rheumatoid arthritis: the effect of patient education, health literacy, and musculoskeletal ultrasound. Biomed Res Int 2015;2015:150658. 26060812 10.1155/2015/150658 PMC4427825

[55] Oshotse C , Zullig LL, Bosworth HB, Tu P, Lin C. Self-efficacy and adherence behaviors in rheumatoid arthritis patients. Prev Chronic Dis 2018;15:E127. 30339772 10.5888/pcd15.180218 PMC6198676

