# Supplementary material for: Quality of life, disease activity and preferences for administration routes in rheumatoid arthritis: a multicentre, prospective, observational study
Source: Rheumatol Adv Pract. 2022 Sep 2;6(3):rkac071. doi: 10.1093/rap/rkac071 (PMC9486987; doi:10.1093/rap/rkac071)
Supplement: rkac071_Supplementary_Data [file rkac071_supplementary_data.docx]

**Supplementary Table S1**. Active medications received by the patients at study time points

| **Medications** | **0-month**  **n (%)** | **3-months**  **n (%)** | **6-months**  **n (%)** | **9-months**  **n (%)** | **12-months**  **n (%)** |
| --- | --- | --- | --- | --- | --- |
| Abatacept | 40 (8.7) | 31 (8.8) | 29 (9) | 27 (9.2) | 23 (7.5) |
| Adalimumab | 46 (10.0) | 36 (10.1) | 25 (7.7) | 27 (9.2) | 28 (9.1) |
| Etanercept | 40 (8.7) | 39 (11) | 28 (8.7) | 30 (10.3) | 35 (11.4) |
| Golimumab | 21 (4.6) | 14 (3.9) | 15 (4.6) | 15 (5.2) | 12 (3.9) |
| İnfliximab | 8 (1.7) | 8 (2.3) | 7 (2.2) | 6 (2.1) | 5 (1.6) |
| Rituximab | 69 (15) | 36 (10.1) | 53 (16.4) | 41 (14) | 53 (17.2) |
| Certolizumab pegol | 44 (9.6) | 34 (9.6) | 30 (9.3) | 32 (11) | 30 (9.7) |
| Tofacitinib | 143 (31.2) | 115 (32.4) | 99 (30.6) | 85 (29.1) | 93 (30.2) |
| Tocilizumab | 48 (10.5) | 42 (11.8) | 37 (11.5) | 29 (9.9) | 29 (9.4) |
| **TOTAL** | 459 (100) | 355 (100) | 323 (100) | 292 (100) | 308 (100) |
| **Missing** | 0 | 104 | 136 | 167 | 151 |

**Supplementary Table S2.** Medication switch during the study period

| **Medication Switch** | **n (%)** |
| --- | --- |
| Yes | 57 (12) |
| Once | 11 (2) |
| Switch and then back to the former medication | 36 (8) |
| More than once | 10 (2) |
| No | 402 (88) |
| Overall Total | 459 (100) |

Supplementary Table S3. EQ-5D and EQ-VAS scores at measurement time points

|  | **Baseline** | | **3-months** | | **6-months** | | **9-months** | | **12-months** | |
| --- | --- | --- | --- | --- | --- | --- | --- | --- | --- | --- |
|  | **EQ-5D** | **EQ-VAS** | **EQ-5D** | **EQ-VAS** | **EQ-5D** | **EQ-VAS** | **EQ-5D** | **EQ-VAS** | **EQ-5D** | **EQ-VAS** |
| N | 459 | 459 | 356 | 356 | 323 | 323 | 292 | 292 | 308 | 308 |
| Mean | 0.341 | 57.7 | 0.622 | 38.2 | 0.645 | 38.7 | 0.699 | 37.4 | 0.688 | 33.9 |
| Standard deviation | 0.376 | 23.6 | 0.303 | 23.8 | 0.325 | 25.5 | 0.297 | 26.3 | 0.294 | 23.7 |
| Minimum | -0.594 | 0 | -0.181 | 0 | -0.429 | 0 | -0.349 | 0 | -0.239 | 0 |
| Maximum | 1.000 | 100 | 1.000 | 100 | 1.000 | 100 | 1.000 | 100 | 1.000 | 100 |
| Shapiro-Wilk (p) | < 0.001 | <0 .001 | <0 .001 | < 0.001 | <0 .001 | <0 .001 | <0 .001 | <0 .001 | <0 .001 | <0 .001 |
| 25th percentile | -0.016 | 50.0 | 0.516 | 20.0 | 0.516 | 20.0 | 0.585 | 20.0 | 0.585 | 15.0 |
| 50th percentile | 0.516 | 60.0 | 0.656 | 40.0 | 0.710 | 40.0 | 0.727 | 30.0 | 0.726 | 30.0 |
| 75th percentile | 0.620 | 80.0 | 0.814 | 50.0 | 0.850 | 50.0 | 1.000 | 51.3 | 0.912 | 50.0 |

Supplementary Table S4. DAS28-4 (ESR) scores at measurement time points

|  | **DAS28-4 (ESR)** | | | | |
| --- | --- | --- | --- | --- | --- |
|  | **Baseline** | **3-months** | **6-months** | **9-months** | **12-months** |
| N | 459 | 356 | 322 | 292 | 308 |
| Mean | 4.99 | 3.45 | 3.31 | 3.02 | 3.01 |
| Standard deviation | 1.17 | 1.31 | 1.24 | 1.19 | 1.23 |
| Minimum | 1.41 | 0.420 | 0.630 | 0.00 | 0.00 |
| Maximum | 8.33 | 7.95 | 7.44 | 6.96 | 7.26 |
| Shapiro-Wilk (p) | 0.888 | 0.035 | <0 .001 | 0.009 | 0.001 |
| 25th percentile | 4.25 | 2.56 | 2.50 | 2.26 | 2.22 |
| 50th percentile | 5.03 | 3.44 | 3.16 | 2.84 | 2.84 |
| 75th percentile | 5.79 | 4.35 | 4.19 | 3.72 | 3.80 |

Supplementary Figure S1. EQ-5D patient responses at (B) 3 months, (C) 6 months, (D) 9 months.

**Supplementary Table S5.** Patient preferences for route of administration at the study visits

|  | **Baseline** | **3-months** | **6-months** | **9-months** | **12-months** |
| --- | --- | --- | --- | --- | --- |
|  | **n (%)** | **n (%)** | **n (%)** | **n (%)** | **n (%)** |
| Oral | 246 (53.6) | 205 (57.6) | 174 (53.9 ) | 154 (52.7 ) | 154 (50.0) |
| Subcutaneous | 125 (27.2 ) | 99 (27.8) | 82 (25.4) | 86 (29.5) | 78 (25.3) |
| Intravenous | 88 (19.2) | 52 (14.6 ) | 67 (20.7 ) | 52 (17.8 ) | 76 (24.7) |
| **TOTAL** | 459 (100.0) | 356 (100.0) | 323 (100.0) | 292 (100.0) | 308 (100.0) |
